# Adequacy of enteral nutritional support in intensive care units does not affect the short- and long-term prognosis of mechanically ventilated patients: a pilot study

**DOI:** 10.5935/0103-507X.20190004

**Published:** 2019

**Authors:** Cecília Flávia Lopes Couto, Ângela Dariano, Cassiano Texeira, Carolina Hauber da Silva, Anelise Bertotti Torbes, Gilberto Friedman

**Affiliations:** 1 Programa de Pós-Graduação em Ciências Pneumológicas, Universidade Federal do Rio Grande do Sul - Porto Alegre (RS), Brasil.; 2 Unidade de Terapia Intensiva, Hospital Moinhos de Vento - Porto Alegre (RS), Brasil.; 3 Santa Casa de Misericórdia de Porto Alegre, Universidade Federal de Ciências da Saúde de Porto Alegre - Porto Alegre (RS), Brasil.

**Keywords:** Critical illness, Critical care, Nutritional support, Enteral nutrition, Energy requirement, Activities of daily living, Respiration, artificial, Intensive care units

## Abstract

**Objective:**

To correlate short-term (duration of mechanical ventilation and length of intensive care unit stay) and long-term (functional capacity) clinical outcomes of patients who reached nutritional adequacy ≥ 70% of predicted in the first 72 hours of hospitalization in the intensive care unit.

**Methods:**

This was a prospective observational pilot study conducted in an 18-bed intensive care unit. A total of 100 mechanically ventilated patients receiving exclusive enteral nutritional support and receiving intensive care for more than 72 hours were included. Patients who never received enteral nutrition, those with spinal cord trauma, pregnant women, organ donors and cases of family refusal were excluded. The variables studied were nutritional adequacy ≥ 70% of predicted in the first 72 hours of hospitalization, length of intensive care unit stay, duration of mechanical ventilation and the ability to perform activities of daily living after 12 months, assessed via telephone contact using the Lawton Activities of Daily Living Scale.

**Results:**

The mean duration of mechanical ventilation was 18 ± 9 days, and the mean intensive care unit length of stay was 19 ± 8 days. Only 45% of the patients received more than 70% of the target nutrition in 72 hours. There was no association between nutritional adequacy and short-term (duration of mechanical ventilation, length of stay in the intensive care unit and mortality) or long-term (functional capacity and mortality) clinical outcomes.

**Conclusion:**

Critically ill patients receiving caloric intake ≥ 70% in the first 72 hours of hospitalization did not present better outcomes in the short term or after 1 year.

## INTRODUCTION

The discrepancy between nutrient prescription and delivery in the acute phase of critical illness is common, which often makes nutritional support suboptimal.^(^^1,2^^)^ Previous research relates low calorie delivery to worse outcomes.^(^^1-4^^)^ Recent studies have shown that permissive underfeeding, for up to 1 week, may be associated with better outcomes.^(^^5,6^^)^

Researchers argue that the apparent benefit of underfeeding may be a misconception resulting from the lack of description of medium- and long-term outcomes. An observational study on the impact of long-term underfeeding has challenged its apparent benefit.^(^^7^^)^ Nevertheless, another clinical study has shown that underfeeding is associated with lower long-term mortality,^(^^5^^)^ and a third study showed no difference in mortality or quality of life.^(^^8^^)^ However, the lack of generalization, the inclusion of patients at low nutritional risk and the methodological issues of these studies are factors that make the interpretation and applicability of these data difficult.^(^^9,10^^)^ It is important to assess how interventions in the intensive care unit (ICU), including nutritional support recommendations, affect critically ill patients in the long term.^(^^11,12^^)^

The quality of life can be affected by the muscle weakness of critically ill patients, as it compromises functional capacity in the medium- and long-term.^(^^13^^)^ Recent studies have shown that muscle weakness may be associated with increasing doses of nutrition in the acute phase of critical illness.^(^^14,15^^)^ The most plausible explanation for the negative impact of early and full feeding is feeding‐induced suppression of autophagy.^(^^16^^)^

The primary objective of this pilot study was to test the hypothesis that nutritional adequacy ≥ 70% of predicted in the first 72 hours of ICU stay in mechanically ventilated patients is associated with short-term clinical outcomes (duration of mechanical ventilation (MV) and length of stay in the ICU). The secondary objectives were to assess ICU mortality and the ability to perform Activities of Daily Living (ADL) 12 months after ICU discharge.

## METHODS

This was a prospective, observational pilot study conducted at an 18-bed medical-surgical ICU at *Complexo Hospitalar Santa Casa de Porto Alegre* and registered with the Ethics Committee (CAAE 14709313.0.0000.5335). Patients undergoing invasive MV and exclusive enteral nutritional support were included if the length of hospital stay was greater than 72 hours. Patients who had never received enteral nutrition, patients with spinal cord trauma, pregnant women and organ donors were excluded. The relatives of all participants signed an informed consent form.

Data related to nutritional support were collected, such as the start of enteral nutrition, time to reach 70% caloric adequacy and total calorie and protein requirements.^(^^5^^)^ Nutritional requirements were calculated according to the unit's protocol, which uses 20-30 kcal per kg of body weight per day. The current weight is used when it is possible to obtain this information from a relative or from a previous hospitalization; otherwise, the weight is estimated using the Chumlea formula.^(^^17^^)^

Regarding the clinical outcomes, data on MV duration, ICU length of stay and ICU mortality were collected. Patients were followed up for 28 days or until hospital discharge (whichever occurred first). After 12 months, telephone contact was made with the responsible family member, and the Lawton Activities of Daily Living Scale was applied to evaluate the individual's functional capacity.^(^^18^^)^ Demographic data and the reason for admission were recorded, and the Acute Physiology and Chronic Health Evaluation (APACHE II) score and the Sequential Organ Failure Assessment (SOFA) score were assessed as disease severity scores.^(^^19,20^^)^

### Statistical analysis

Quantitative variables are described as the mean and standard deviation or as the median and interquartile range. Qualitative variables are described as absolute and relative frequencies. Student's *t* test and one*-*way ANOVA were applied to compare means. In case of asymmetry, the Mann-Whitney and Kruskal-Wallis tests were used. For comparison of proportions, the chi-squared test, Pearson's correlation or Fisher's exact test was applied. To complement these analyses, the adjusted residuals test was used. The associations between continuous variables were evaluated by the Pearson (r) or Spearman (rs) correlation coefficients.

The sample size was calculated based on the study of Castro et al.,^(^^21^^)^ which showed a reduction of 9 days in ICU stay relative to the calories prescribed in the first 72 hours of hospitalization. For our study, a minimum of 60 patients to be included was estimated, considering a normal distribution for ICU length of stay, with a significance level of 5% and a power of 90%. Thus, a minimum of 60 patients to be included was defined.

The significance level adopted was 5% (p ≤ 0.05), and the analyses were performed in the Statistical Package for Social Science (SPSS), version 21.0 (IBM Corp., Armonk, New York, USA).

## RESULTS

The demographic and clinical characteristics are described in table 1.

**Table 1:** Sample characteristics

Variable	n = 100	Full feeding ≥ 70% n = 45	Full feeding < 70% n = 55
Age (years)	64 ± 16	63 ± 17	65 ± 16
Gender (male/female)	56/44		
APACHE II	21 ± 7	22 ± 8	20 ± 7
SOFA	7 ± 3	7 ± 3	6 ± 3
Patients with ICU stay ≥ 28 days	33	12	21
Tracheostomized	48	20	28
Diagnosis on admission			
Cardiopathy	11	4	7
Hepatopathy	5	2	3
Infection	17	10	7
Respiratory disease	28	14	14
Cancer	15	7	8
Surgeries	18	5	13
Other	6	3	3
ICU outcome (n)			
Discharge	27	13	14
Stay > 28 days	33	12	21
Calories prescribed	1,621 ± 203	1,554 ± 203	1,677 ± 187*
Calories/kg	26 ± 3	27 ± 4	26 ± 2
Protein (g)	91 ± 17	86 ± 18	96 ± 16
Protein (g/kg)	1.46 ± 0.12	1.46 ± 0.12	1.47 ± 0.11

APACHE II - Acute Physiology and Chronic Health Evaluation II; SOFA - Sequential Organ Failure Assessment; ICU - intensive care unit.

* p = 0.002. Results are expressed as the mean ± standard deviation or n.

**Figure 1:**
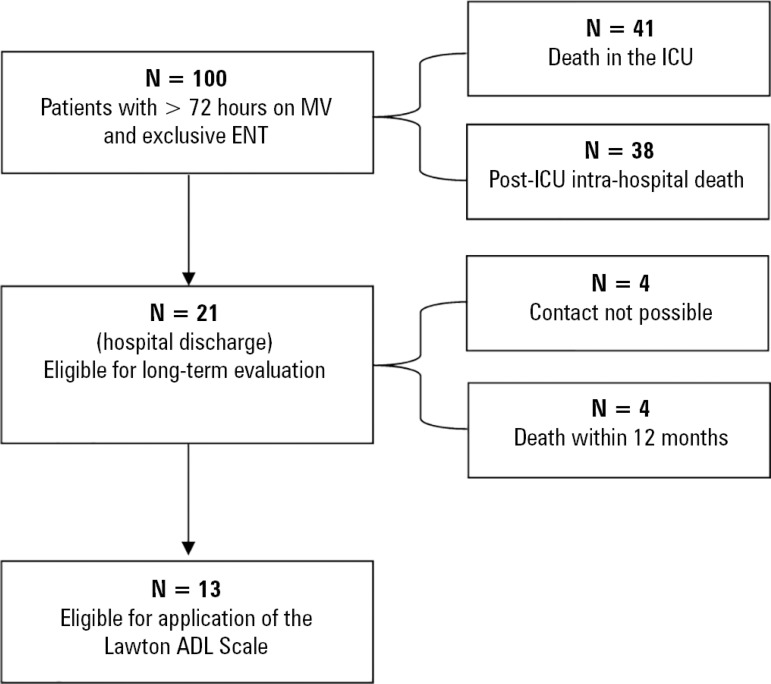
Data collection flowchart. MV - mechanical ventilation; ENT - enteral nutrition therapy; ICU - intensive care unit; ADL - Activities of Daily Living.

A total of 100 patients were included during the study period. Most patients (55%) did not reach 70% nutritional adequacy. There were 79 intrahospital deaths, plus an additional 4 during the 12-month follow-up. Four patients were lost to follow-up due to the impossibility of contacting family members via telephone. Therefore, 13 patients were evaluated after 12 months of follow-up (Figure 1). There was no association between nutritional adequacy in the first 72 hours and short- or long-term clinical outcomes (Table 2).

**Table 2:** Association between nutritional adequacy and short- and long-term outcomes

Variable	Full feeding on admission ≥ 70% adequacy		p value
	Yes (n = 45)	No (n = 55)	
Short-term outcomes (up to 28 days in the ICU)			
MV duration (days)	17.2 ± 8.7	18.8 ± 8.6	0.372
ICU stay (days)	18.2 ± 8.0	19.9 ± 7.9	0.298
ICU mortality	21 (46.7)	20 (36.4)	0.402
Long-term outcomes			
Functional capacity	14 (8 - 19) (n = 7)	6 (0 - 16) (n = 6)	0.445
Mortality at 1 year after ICU discharge	3 (30.0) (n = 10)	1 (14.3) (n = 7)	0.603

ICU - intensive care unit; MV - mechanical ventilation; SD - standard deviation; P25 - 25^th^ percentile; P75 - 75^th^ percentile.

* Significant association by the adjusted residuals test at 5% significance. The results are expressed as the mean ± standard deviation, n (%) or median (P25 - P75).

## DISCUSSION

The main finding of this pilot study was that nutritional adequacy (caloric target > 70%) in the first 72 hours of ICU stay is not associated with improvement of short-term outcomes (duration of MV, length of ICU stay and mortality). In addition, the ability to perform ADL 1 year after discharge from the ICU does not appear to have been influenced by nutritional adequacy.

Studies have suggested an association between a negative energy balance and worse clinical outcomes (duration of MV, length of stay in the ICU and hospital) and ICU mortality, suggesting that adequate caloric intake could be associated with better clinical outcomes.^(^^2,22-25^^)^ This topic has been extensively debated; however, recent meta-analyses do not support this hypothesis^(^^26,27^^)^ and do not show a reduction in the length of ICU stay, length of hospital stay, duration of MV, or mortality rate. Our results were similar to those found in the EDEN study,^(^^28^^)^ which found no difference in the clinical outcomes of mechanically ventilated patients who were provided reduced caloric supply in the first 6 days of ICU stay. The sample size for the present study was based on the study of Castro et al.,^(^^21^^)^ who found a 9-day reduction in ICU length of stay. We speculate that the effect size in that study indicates that there was a difference in severity that was not revealed by the evaluation used and that acceptance of feeding was therefore more effective and associated with a better prognosis.

Few studies have evaluated the consequences of nutritional adequacy in the acute phase of critical illness on long-term outcomes.^(^^11^^)^ Wei et al.^(^^7^^)^ evaluated the relationship between nutritional adequacy and short- and long-term clinical outcomes, including 6-month survival and quality of life in critically ill patients requiring prolonged MV. The authors suggest an important relationship between full nutritional support in the first week of hospitalization and higher survival at 6 months of follow-up. Our study does not replicate these findings, since 6 of the 13 patients who completed the functional capacity assessment at 12 months and who did not reach the caloric target were no longer dependent.

Our study has several limitations, such as its observational nature, small number of patients studied by convenience (not all admitted patients were screened) to evaluate long-term outcomes, no collection of data on nutrient prescription and delivery, lack of comparison of functional capacity before and after ICU admission and the lack of evaluation of patient nutritional status or other nutritional risk scores. The questionnaire was applied only at 12 months after discharge from the unit, and there were inevitable patient losses due to changed telephone numbers. A very severely ill population was selected, and all patients were mechanically ventilated, had high mean APACHE II scores and had almost 7 organ dysfunctions on average. The high mortality limits the interpretation of the short-term outcomes but mainly affects the long-term outcomes due to the small number of analyzed patients.

## CONCLUSION

The results of this pilot study show that critically ill patients receiving a caloric intake ≥ 70% in the first 72 hours of hospitalization do not present better short-term outcomes. Secondarily, nutritional adequacy does not appear to have positively influenced long-term functional capacity.
